# Rosmarinic Acid Attenuates Rotenone-Induced Neurotoxicity in SH-SY5Y Parkinson’s Disease Cell Model through Abl Inhibition

**DOI:** 10.3390/nu14173508

**Published:** 2022-08-26

**Authors:** Xiao Han, Bing Han, Yue Zhao, Gang Li, Tian Wang, Jie He, Wenxiao Du, Xiaolin Cao, Jing Gan, Zhenhua Wang, Wei Zheng

**Affiliations:** 1 Center for Mitochondria and Healthy Aging, College of Life Sciences, Yantai University, Yantai 264005, China; 2 Shandong Hongli Medical Animal Experimental Research Co., Jinan 250000, China; 3 School of Pharmacy, Yantai University, Yantai 264005, China; 4 College of Life Science, Yantai University, Yantai 264005, China

**Keywords:** rosmarinic acid, Parkinson’s disease, mitochondrial function, Abl tyrosine kinase, rotenone

## Abstract

Rosmarinic acid (RA) is a natural polyphenolic compound with antioxidative property. With the present study, we aimed to evaluate the neuroprotective role of RA on Parkinson’s disease using rotenone induced SH-SY5Y cell model of Parkinson’s disease, the underlying mechanism of action of RA was also investigated. Cell viability, cell morphology, apoptosis, signaling protein phosphorylation and expression, cellular reactive oxygen species (ROS) production, ATP content, and mitochondrial membrane potential were tested in SH-SY5Y cells. RA showed a neuroprotective effect in a rotenone-induced SH-SY5Y cell model of Parkinson’s disease with dose-dependent manner, it reduced cell apoptosis and restored normal cell morphology. RA not only decreased levels of α-synuclein and Tau phosphorylation but also elevated the contents of AMPK phosphorylation, Akt phosphorylation, and PGC-1α. RA restored the reduced mitochondrial membrane potential and ATP content as well as inhibited rotenone-induced ROS overproduction. Further findings demonstrated that the neuroprotective role of RA was partially due to the inhibition of Abl tyrosine kinase. RA treatment suppressed the hyperphosphorylation of Abl Y412 and CrkII Y221 induced by rotenone. Nilotinib, a specific inhibitor of Abl, elicited a similar neuroprotective effect as that of RA. The present study indicates that RA has a property of neuroprotection against rotenone, and the neuroprotective effect is partially attributed to the inhibition of Abl.

## 1. Introduction

Parkinson’s disease (PD) is the second most prevalent neurodegenerative disease. There are currently more than five million PD patients worldwide. Up to date, no curing treatment is available [1]. The main characteristics of PD are the degeneration of dopaminergic neurons in the substantia nigra pars compacta and the lack of dopamine in the striatum [2], which result in the clinical features including tremor, bradykinesia, and muscle stiffness [3]. A few molecular mechanisms have been identified to be responsible for the neuronal cell death in PD, including mitochondrial dysfunction, increased iron content, and oxidative stress. Previous studies showed an increase of oxidative stress in the cerebrospinal fluid of PD patients and suggested that excess formation of free radicals was involved in the progression of PD [4]. Therefore, several research studies were carried out to investigate the roles of antioxidative agents on treating PD animal or cell models [5,6].

**Figure 1:**
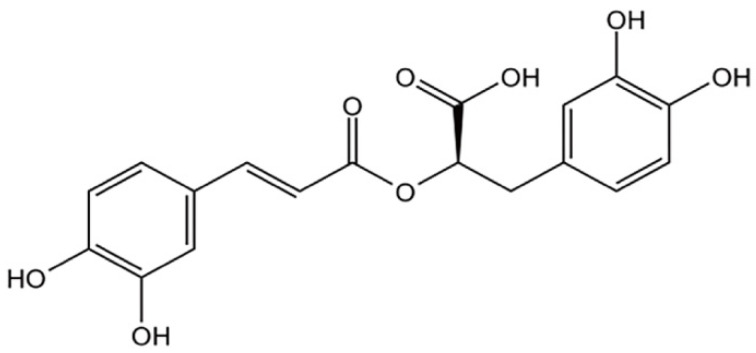
Chemical structure of rosmarinic acid (RA).

Rosmarinic acid (RA) is a natural polyphenolic compound that exists in several kinds of plants including Lamiaceae and Boraginaceae families [7] (Figure 1). It has antiviral, antibacterial, anti-inflammatory, and antioxidative properties [8]. A PD cell model study with SH-SY5Y showed that RA activated antioxidant enzyme heme oxygenase-1 to suppress reactive oxygen species (ROS) production and cell death induced by H_2_O_2_ exposure [9]. RA also reduced the oxidative stress and neurotoxic effects induced by 6-hydroxydopamine (6-OHDA) and reversed the mitochondrial membrane potential reduction in 6-OHDA challenged cells [10]. Another study further proved the neuroprotective effect of RA by re-establishing the mitochondrial complex I function and recovering the dopamine content level and cell viability in 1-methyl-4-phenylpyridinium (MPP+) exposed MES23.5 dopaminergic cells [11]. In PD animal model, RA was demonstrated to protect against 1-methyl-4-phenyl-1,2,3,6-tetrahydropyridine (MPTP) induced neurotoxicity in zebrafish embryos [6]. These studies indicated RA being a potential compound for PD treatment.

Rotenone is a natural compound present in plants including Tephrosia and Lonchocarpus [12]. Since the rotenone challenged animal models display two typical characteristics of PD, Lewy bodies in the substantia nigra neurons and degeneration of these cells [13], rotenone exposed animal or cell model gives one of the best experimental models for research on neuroprotective therapies for PD [14,15,16]. In neuronal cells, rotenone has an inhibitory effect on mitochondrial complex I and mediates α-synuclein aggregation, it causes mitochondrial dysfunction and overproduction of ROS, which ultimately induces neuronal cell death [17,18,19]. Up to date, the molecular mechanism of action of RA protecting neuronal cells has not been fully elucidated, and the neuroprotective role of RA on rotenone-exposed cells has not been evaluated.

Abelson tyrosine kinase (Abl) is a nonreceptor tyrosine kinase. Upon being activated by oxidative stress, Abl phosphorylates α-synuclein and promotes its accumulation, aggregation, and plays an essential role in neuron degeneration [20]. Abl inhibition rescued mitochondrial function reduction and dopaminergic neuronal degeneration in several mouse PD models [20]. A potent Abl inhibitor, nilotinib, was first developed to treat chronic myeloid leukemia [21]. Later study showed that nilotinib protected dopaminergic neurons in MPTP-induced preclinical mice model of PD [22].

The aim of the current study was to explore the neuroprotective effects of RA on rotenone challenged SH-SY5Y cell model of PD. Furthermore, the role of Abl in the neuroprotective mechanism of RA against rotenone was also assessed.

## 2. Materials and Methods

### 2.1. Cell Culture

Human neuroblastoma SH-SY5Y cells were purchased from Shanghai Cell Bank (Shanghai, China). The cells were cultured in DMEM media (Gibco, Waltham, MA, USA) with 10% (*v*/*v*) fetal bovine serum (FBS) (TIANHANG, Zhejiang, China), 100 U/mL penicillin, and 100 mg/mL streptomycin (Gibco, Waltham, MA, USA). Cells were maintained at 37 °C in a saturated humidity atmosphere containing 95% air and 5% CO_2_. Cells grew to around 70% confluences before they were used for experiments.

### 2.2. Cell Viability Assay and Morphological Observation

Cell viability was evaluated using 3-(4,5-dimethylthiazol-2-yl)-2,5-diphenyltetrazolium bromide (MTT) assay. In previous research, RA was pre-administered to animals or cells for different lengths of time before they were challenged with the disease-modeling reagents. RA pretreatment for 2 h protects against MPTP-induced neurotoxicity in zebrafish embryos dopaminergic neurons [6]. Pretreatment for 30 min with RA protects SK-N-SH cells against MPTP induced toxicity [8]. Carnosic acid is a natural antioxidant compound derived from rosemary from which rosmarinic acid was also derived [23]. 12 h pretreatment with carnosic acid has neuroprotective effect on SH-SY5Y cells against 6-OHDA induced neurotoxicity [23]. Based on these data, a medium time length, 3 h, was chosen as the RA pretreatment time length before SH-SY5Y cells were exposed to rotenone. SH-SY5Y cells were exposed to 0, 0.1, 1, 10, 50, 100, and 200 µM rotenone for 24 h at 37 °C for the rotenone toxicity evaluation. SH-SY5Y cells were pretreated with rosmarinic acid (RA, Sigma-Aldrich, St. Louis, MO, USA) before they were then either exposed to 50 µM rotenone, or 0, 1, 10, or 100 µM RA together with rotenone, or 30 nM nilotinib with rotenone, or RA alone, or nilotinib alone for 24 h at 37 °C. Then, 10 µL of MTT (5 mg/mL) was added to each well in a 96-well plate and incubated for 4 h. The insoluble blue formazan was then solubilized with 100 µL/well dimethyl sulfoxide (DMSO), and optical density (OD) values of the mixture were measured at 595 nm with a SpectraMax Paradigm Multi-Mode Microplate Reader (Molecular Devices, Sacramento, CA, USA). Each sample in MTT assay was measured at least in triplicate. For cell morphology analysis, the cells were observed and photographed using a Leica Microsystems microscope (Leica Microsystems CMS GmbH, Wetzlar, Germany) after exposed to 50 µM rotenone and 100 µM RA.

### 2.3. Apoptosis Assay

SH-SY5Y cells were seeded in 6-well plates and then exposed to 50 µM rotenone and 100 µM RA for 24 h before they were harvested by trypsin solution to produce a single cell suspension. The cells were pelleted by centrifugation and washed twice with PBS to remove trypsin. The cell suspension was then prepared and stained with a YF488-Annexin V and PI Cell Apoptosis Assay Kit (Biorigin, Beijing, China) according to the instruction. The stained cells were analyzed using a ACEA Novocyte flow cytometer (ACEA Biosciences, San Diego, CA, USA) in combination with Novo Express software.

### 2.4. Western Blot Assay

For Western blot experiments, SH-SY5Y cells were treated as indicated, in the presence or absence of 50 µM rotenone, 100 µM RA, and 30 nM of Nilotinib. Cells were treated for 90 min for the analysis of phosphorylation levels and for 24 h for the analysis of protein levels. Cells were then rinsed with PBS before they were lysed with RIPA buffer (150 mmol/L NaCl, 50 mmol/L Tris–HCl (pH 7.4), 100 mg/mL Phenylmethyl Sulphonyl Fluoride (PMSF), 1% NP-40, 0.1% sodium dodecyl sulfate (SDS)) for 15 min. The cells were scraped and collected before they were centrifuged at 13,000× *g* for 15 min at 4 °C. The supernatants were collected for the following analysis. A bicinchoninic acid (BCA) Protein Assay Kit was then used to measure the protein concentration in the collected cell lysates. The cell lysates were mixed with loading buffer and boiled for 5 min. 30 µg protein from each sample was used for SDS polyacrylamide gel electrophoresis (SDS-PAGE) analysis (Miniprotean; Bio-Rad). Proteins separated on the gel were then transferred onto Immunobilon-P polyvinylidene difluoride (PVDF) membranes (Millipore, Bedford, MA, USA). 5% BSA (Sigma-Aldrich, St. Louis, MO, USA) in TBS Tris-buffered saline (68 mmol/L NaCl, 10 mmol/L Tris-base, pH 7.5) containing 0.1% Tween 20 was used for 1 h membrane blocking at room temperature. Recommended concentration of primary antibodies were incubated together with the blocked membrane at 4 °C overnight. Membranes were washed and incubated with peroxidase conjugated antibodies for 1 h at room temperature. After the washing step, bound antibodies were visualized using a 5200 Multi Luminescent image analyzer (Tanon Science & Technology Co., Ltd., Shanghai, China) and quantified with Image pro plus 6.0 software. The antibodies used in this study were against: Tau (Abcam, Cambridge, UK), Phospho-Tau Ser404 (Abcam, Cambridge, UK), AMPK (Cell Signaling Technology, Danvers, MA, USA), Phospho-AMPK Thr172 (Cell Signaling Technology, Danvers, MA, USA), Akt (Cell Signaling Technology, Danvers, MA, USA), Phospho-Akt Ser473 (Cell Signaling Technology, Danvers, MA, USA), c-Abl (Cell Signaling Technology, Danvers, MA, USA), Phospho-c-Abl Tyr412 (Cell Signaling Technology, Danvers, MA, USA), Phospho-CrkII Tyr221 (Cell Signaling Technology, Danvers, MA, USA), α-synuclein (Cell Signaling Technology, Danvers, MA, USA), PGC-1α (Cell Signaling Technology, Danvers, MA, USA), β-actin (Cell Signaling Technology, Danvers, MA, USA). The secondary antibody used was horseradish peroxidase (HRP) Goat anti-Rabbit immunoglobulin G (IgG).

### 2.5. Measurement of Mitochondrial Membrane Potential

Fluorescent probe JC-1 (Thermo Fisher Scientific, Waltham, MA, USA) was used to evaluate the mitochondrial membrane potential. After being exposed to 50 µM rotenone, 100 µM RA or 30 nM nilotinib for 12 h, cells were incubated with JC-1 staining solution (5 µg/mL) for 30 min at 37 °C. A single cell suspension was then obtained by cell trypsinization. The cells were collected by centrifugation before they were rinsed with PBS. The cell-bound JC-1 fluorescent signal was analyzed using a ACEA Novocyte flow cytometer (ACEA Biosciences, San Diego, CA, USA) in combination with Novo Express software. The mitochondrial membrane potential of SH-SY5Y cells was calculated as the ratio of red fluorescence to green fluorescence.

Cells were cultured on coverslips at a density of 1 × 10^5^ cells/mL in 24 well plates for 24 h. After the indicated treatments, the cells were stained with JC-1 for 30 min at 37 °C. Fluorescence microscope (Leica Microsystems CMS GmbH, Wetzlar, Germany) was used to capture fluorescence images.

### 2.6. ROS Assay

After being exposed to 50 µM rotenone, 100 µM RA, or 30 nM nilotinib for 1 h, the cells were co-incubated with DMEM containing 10 µM of DCFH-DA for 30 min at 37 °C. A single cell suspension was then obtained using cell trypsinization. The cells were collected using centrifugation before they were rinsed with PBS. The cell-bound DCFH-DA fluorescent signal was analyzed using a ACEA Novocyte flow cytometer (ACEA Biosciences, San Diego, CA, USA) in combination with Novo Express software.

Cells were cultured on coverslips at a density of 1 × 10^5^ cells/mL in 24 well plates for 24 h. After the indicated treatments, cells were coincubated with DMEM containing 10 µM of DCFH-DA for 30 min at 37 °C. A fluorescence microscope (Leica Microsystems CMS GmbH, Wetzlar, Germany) was used to capture fluorescence images.

### 2.7. ATP Assay

ATP content in SH-SY5Y cells was measured using a luciferase-based luminescence enhanced ATP assay kit (Beyotime, Shanghai, China). Cells were washed with ice-cold PBS before they were lysed with 100 µL ice-cold ATP releasing buffer. The cell lysates obtained were centrifuged at 12,000× *g* for 5 min at 4 °C. The supernatants were incubated with the ATP testing working solution provided by the kit. ATP content in cell lysates was determined using a SpectraMax Paradigm Multi-Mode Microplate Reader (Molecular Devices, Sacramento, CA, USA).

### 2.8. Statistical Analyses

All data were expressed as the Mean ± SEM. Analysis was performed using GraphPad Prism software 8.0. The data were analyzed using one-way ANOVA followed by Tukey’s post hoc test. Statistical significance was defined as *p* < 0.05.

## 3. Results

### 3.1. RA Restored Cell Viability and Morphology in Rotenone Challenged SH-SY5Y Cells

**Figure 2:**
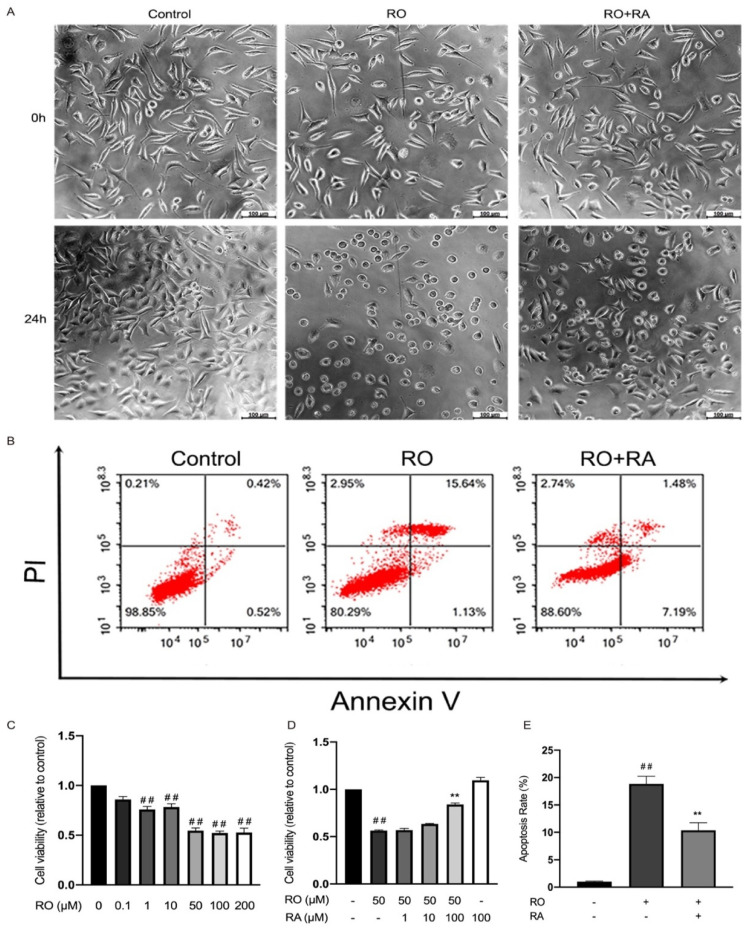
Rosmarinic acid protected SH-SY5Y cells from the toxicity effect of rotenone (RO). (**A**) Cell morphology microscopic photograph of SH-SY5Y cells in the absence or presence of 50 µM rotenone and 100 µM RA. Scale bar = 100 µm. (**B**) Cell apoptosis evaluated with flow cytometry in the absence or presence of rotenone and RA. (**C**) Bar graph quantification analysis of SH-SY5Y cell viability after being exposed to the indicated concentrations of rotenone evaluated with MTT assay. (**D**) Bar graph quantification analysis of SH-SY5Y cell viability in the absence and presence of indicated concentration of rotenone and RA evaluated with MTT assay. (**E**) Bar graph quantification analysis of SH-SY5Y cell apoptosis rate in the absence or presence of rotenone and RA evaluated with flow cytometry. All data were expressed as the Mean ± SEM of at least three independent experiments. ## *p* < 0.01 compared with the control group, ** *p* < 0.01 compared with the rotenone group.

To investigate the role of RA in protecting neurons against the toxic effect of rotenone, SH-SY5Y cell viability and morphology were tested in the absence and presence of rotenone and RA. The results showed that 1–200 µM rotenone significantly reduced cell viability (Figure 2C). Cotreatment with rotenone and increasing concentration of RA led to gradual recovery of cell viability (Figure 2D). RA at a concentration of 100 µM significantly rescued SH-SY5Y cells from the toxic effect of rotenone (Figure 2A,D).

### 3.2. RA Reduced Cell Apoptosis Rate in Rotenone Challenged SH-SY5Y Cells

Apoptosis analysis showed that 24 h rotenone exposure led to increased cell apoptosis rate, while RA treatment significantly reduced cell apoptosis induced by rotenone (Figure 2B,E).

### 3.3. RA Suppressed Tau Phosphorylation and α-Synuclein Expression Elevated by Rotenone

**Figure 3:**
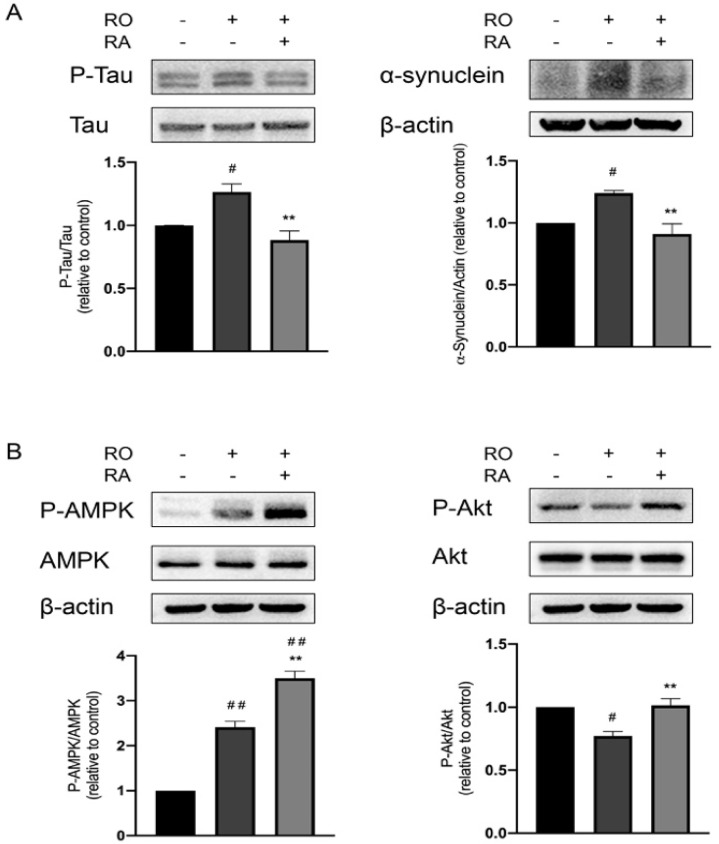
RA treatment modulated the protein level of α-synuclein, the phosphorylation level of Tau Ser404, AMPK Thr172, and Akt Ser473. (**A**) Representative Western blot photographs of the phosphorylation level of Tau, protein levels of α-synuclein, Tau, and β-actin, and the corresponding bar graph quantification analyses of P-Tau relative to Tau protein level and α-synuclein relative to β-actin protein level. (**B**) Representative Western blot photographs of P-AMPK, P-Akt, AMPK, Akt, and β-actin levels, and the corresponding bar graph quantification analyses of P-AMPK relative to AMPK and P-Akt relative to Akt. All data were expressed as the mean ± SEM of three independent experiments. # *p* < 0.05, ## *p* < 0.01 compared with the control group, ** *p* < 0.01 compared with the rotenone group.

Phosphorylation of Tau at Ser404 and accumulation of α-synuclein are considered to be the indicators of PD and induce neurodegeneration [24]. To analyze the molecular mechanism of RA’s neuroprotective effect, Ser404 phosphorylation of Tau and α-synuclein protein level in SH-SY5Y cells were examined. Rotenone exposure increased Tau phosphorylation and α-synuclein level. RA significantly suppressed the elevated Tau phosphorylation and α-synuclein level induced by rotenone (Figure 3A).

### 3.4. RA Rescued Rotenone Induced P-Akt Reduction and Further Promoted the Phosphorylation of AMPK Thr172

AMPK kinase plays important roles in neuronal protection in PD, Thr172 is located in the activation loop of AMPK, and its phosphorylation is required for AMPK activation [25]. Akt signaling is critical for cell survival in PD [26]. Results in Figure 3B showed that rotenone exposure caused upregulation of AMPK Thr172 phosphorylation, while treatment of RA further elevated AMPK phosphorylation. Rotenone exposure also downregulated Akt phosphorylation, and RA recovered Akt phosphorylation.

### 3.5. RA and Nilotinib Reduced Abl Y412 and CrkII Y221 Phosphorylation Elevated by Rotenone and Restored SH-SY5Y Cell Viability under Rotenone Treatment

**Figure 4:**
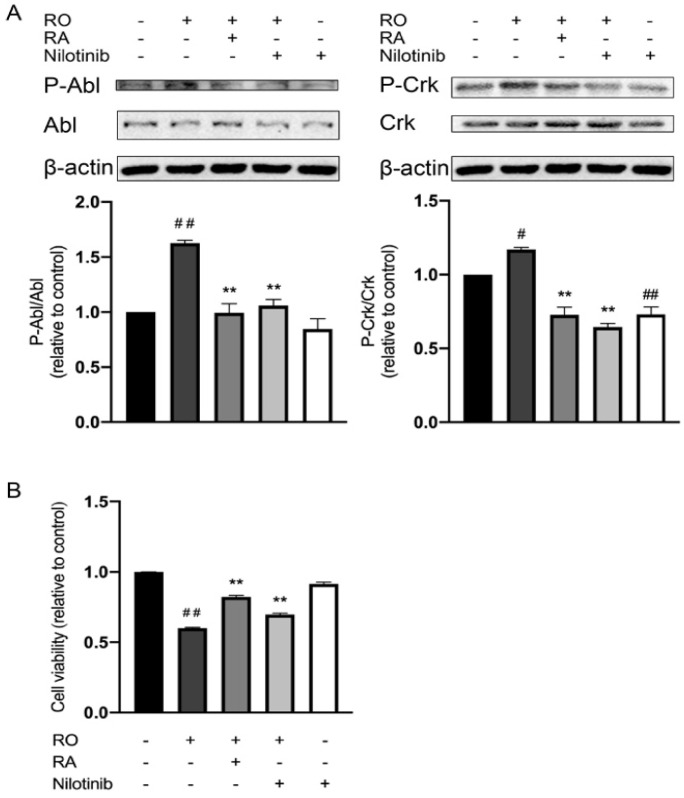
RA and nilotinib reduced Abl Tyr412 and CrkII Tyr221 phosphorylation induced by rotenone and restored SH-SY5Y cell viability under rotenone treatment. (**A**) Representative Western blot photographs of the phosphorylation level of Abl Tyr412, CrkII Tyr221, protein levels of Abl, CrkII and β-actin, and the corresponding bar graphs of quantification analyses of P-Abl relative to Abl protein and P-CrkII relative to Crk protein. (**B**) Bar graph quantification analysis of SH-SY5Y cell viability in the absence and presence of 50 µM rotenone, 100 µM RA, and 30 nM nilotinib evaluated with MTT assay. All data were expressed as the mean ± SEM of three independent experiments. # *p* < 0.05, ## *p* < 0.01 compared with the control group, ** *p* < 0.01 compared with the rotenone group.

Tyrosine kinase Abl plays an essential role in neurodegeneration. CrkII is a well-known substrate of Abl and can be phosphorylated at Tyr221 by Abl [27]. To investigate if Abl kinase is involved in RA-mediated neuroprotective effect against rotenone, cellular phosphorylation of Abl Y412 and CrkII Y221 were evaluated in the presence and absence of rotenone, RA and Abl specific inhibitor, nilotinib. Rotenone exposure induced increases in Abl Y412 and CrkII Y221 phosphorylation, while treatment with nilotinib or RA significantly reduced Abl and CrkII phosphorylation (Figure 4A). As shown in Figure 4B, rotenone reduced the SH-SY5Y cell viability, and RA or nilotinib treatments increased cell viability level in the rotenone-exposed cells.

### 3.6. Abl Inhibition and RA Increased PGC-1α Expression, Akt, and AMPK Phosphorylation under Rotenone Exposure

**Figure 5:**
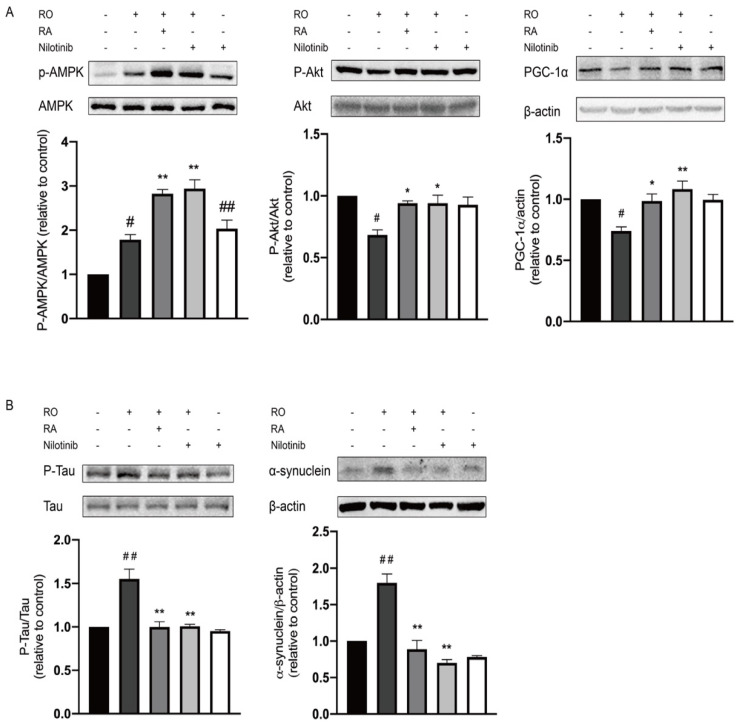
Abl inhibition modulated the protein level of α-synuclein, PGC-1α, and the phosphorylation level of Tau, AMPK, and Akt. (**A**) Representative Western blot photographs of the phosphorylation level of AMPK Thr172, Akt Ser473, and protein level of PGC-1α, AMPK, Akt, and β-actin, and the corresponding bar graphs quantification analyses of P-AMPK relative to AMPK, P-Akt relative to Akt and PGC-1α relative to β-actin. (**B**) Representative Western blot photographs of the phosphorylation level of Tau Ser404, the protein level of α-synuclein, Tau, and β-actin, and the corresponding bar graphs quantification analyses of P-Tau relative to Tau and α-synuclein relative to β-actin. All data were expressed as the mean ± SEM of three independent experiments. # *p* < 0.05, ## *p* < 0.01 compared with the control group, * *p* < 0.05, ** *p* < 0.01 compared with the rotenone group.

Results in Figure 5A showed that rotenone exposure downregulated Akt phosphorylation, PGC-1α expression, and upregulated AMPK phosphorylation. RA and Abl inhibition increased Akt phosphorylation and PGC-1α expression level and further increased AMPK phosphorylation.

### 3.7. Abl Inhibition Reduced Tau Phosphorylation and α-Synuclein Expression Level Elevated by Rotenone

To investigate the mechanism of the neuroprotective effect of RA, Tau Ser404 protein phosphorylation, and α-synuclein level in SH-SY5Y cells were examined. Like RA, nilotinib significantly reduced the elevation of Tau phosphorylation and α-synuclein expression induced by rotenone exposure, while nilotinib alone did not lead to any significant changes in cells in the absence of rotenone (Figure 5B).

### 3.8. Abl Inhibition and RA Suppressed Rotenone-Induced ROS Overproduction

**Figure 6:**
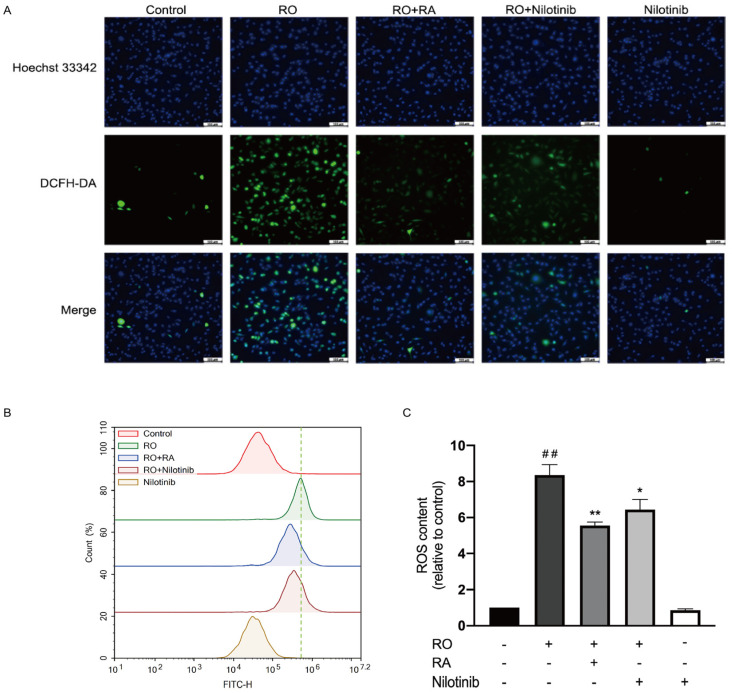
RA and Abl inhibition suppressed rotenone induced ROS production. (**A**) SH-SY5Y cells were exposed to rotenone, RA, and nilotinib for 1 h; then, cells were incubated with 10 µM DCFH-DA for 30 min, images were acquired using fluorescence microscope. Scale bar = 100 µm. (**B**) After treatments indicated, DCFH2-DA fluorescence intensity was evaluated using flow cytometry. (**C**) Bar graph quantification analysis of ROS level evaluated with flow cytometry. All data were expressed as the mean ± SEM of three independent experiments. ## *p* < 0.01 compared with the control group, * *p* < 0.05, ** *p* < 0.01 compared with the rotenone group.

ROS production was measured in the cells. ROS level was markedly increased upon rotenone exposure. RA or nilotinib significantly reduced the ROS production induced by rotenone. Treatment with nilotinib alone did not lead to any significant changes of ROS production when compared with that of the control group (Figure 6A–C).

### 3.9. Abl Inhibition and RA Restored Mitochondrial Membrane Potential and ATP Content in Rotenone Exposed SH-SY5Y Cells

**Figure 7:**
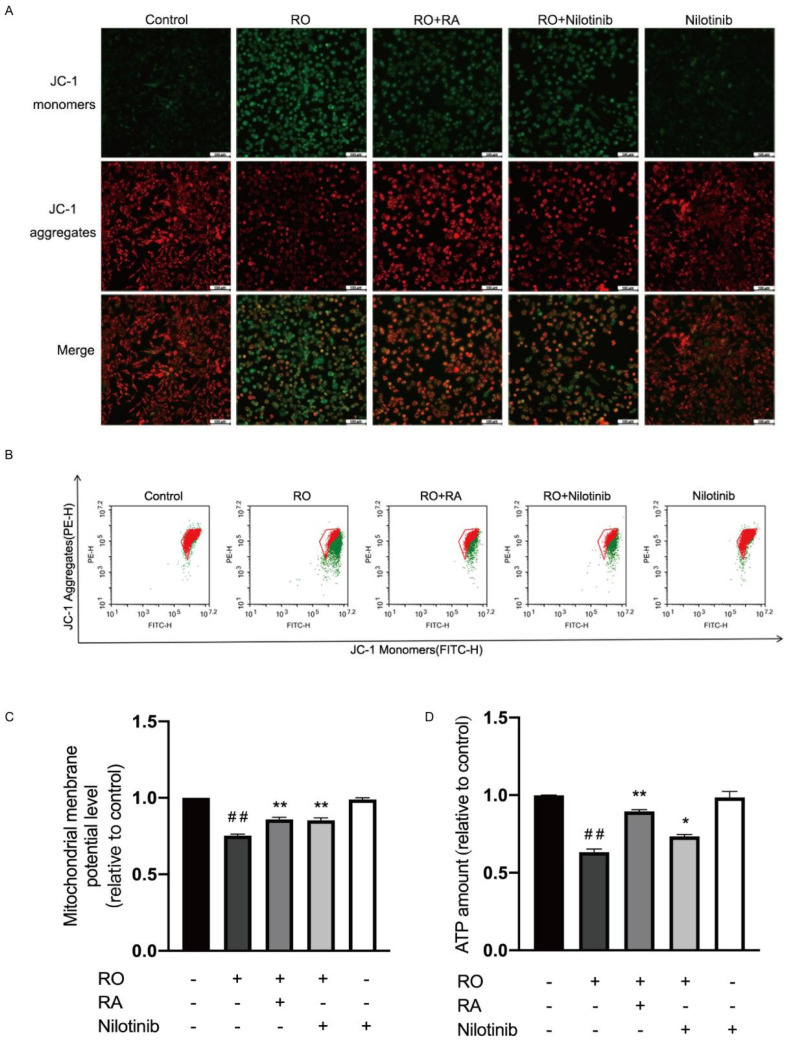
Abl inhibition and RA restored mitochondrial membrane potential and ATP content in rotenone exposed SH-SY5Y cells. (**A**) Cells were exposed to rotenone, RA, and nilotinib for 12 h, the cells were then stained with JC-1 for 30 min, images were acquired using fluorescence microscope. Scale bar = 100 µm. (**B**) After treatments, JC-1 fluorescent signal was detected using flow cytometry (Green: JC-1 monomers, Red: JC-1 aggregates). (**C**) Bar graph quantification analysis of mitochondrial membrane potential evaluated with flow cytometry. (**D**) Bar graph quantification analysis of cellular ATP level measured with luminescence microplate reader. All data were expressed as the mean ± SEM of three independent experiments. ## *p* < 0.01 compared with the control group, * *p* < 0.05, ** *p* < 0.01 compared with the rotenone group.

Mitochondrial membrane potential in SH-SY5Y cells was measured. After rotenone exposure, mitochondrial membrane potential was reduced. RA or nilotinib treatment significantly restored the mitochondrial membrane potential in rotenone exposed cells (Figure 7A–C). Treatment with nilotinib alone did not lead to any significant changes of mitochondrial membrane potential when compared with control. Figure 7D showed the cellular ATP content measured. Rotenone reduced ATP content of the cells. RA or nilotinib treatment significantly restored the reduced ATP content. Nilotinib alone did not lead to any significant changes of ATP amount when compared with control.

## 4. Discussion

In the present study, we evaluated the neuroprotective role of RA in the rotenone challenged SH-SY5Y model and the corresponding molecular mechanism of actions of RA. We demonstrated that RA protected SH-SY5Y cells against rotenone by restoring mitochondrial membrane potential, cellular ATP content and suppressing ROS overproduction. The mechanism of action of RA involves the regulation of Akt, AMPK, Tau phosphorylation, and α-synuclein as well as PGC-1α protein expression. RA exerted this neuroprotective effect partially by attenuating Abl kinase activity.

Rotenone exposure induces cell apoptosis by inhibiting mitochondrial respiratory chain complex I and increasing ROS production in HL60 and HT1080 cell lines [18]. Superoxide dismutase and antioxidants inhibit rotenone-induced apoptosis [18]. In the cell apoptosis analysis of our study, apoptosis rate was calculated as the percentage of Annexin V staining positive cells. The result showed that 50 µM rotenone markedly increased the apoptosis rate of SH-SY5Y cells. RA, as an antioxidant, also significantly reduced rotenone-induced cell apoptosis. Microscopic observation showed that SH-SY5Y cell morphology changed obviously after rotenone exposure. Rotenone exposure made cells become round, RA restored the normal shape of the cells. Cell viability was reduced by rotenone, and 100 µM RA significantly rescued cell viability in the presence of rotenone. These findings indicated a neuroprotective function of RA against rotenone. In the substantia nigra pars compacta of brain, metabolism of dopamine leads to the generation of reactive oxygen species (ROS) including hydrogen peroxide and hydroxyl radicals. This makes this dopamine rich areas particularly vulnerable to oxidative stress [28]. Thus, oxidative stress plays an important role in mediating neuronal cell death in PD [29]. Oxidative stress disrupts the activity of mitochondrial complex I. Loss of mitochondrial complex I activity in the dopaminergic neurons is considered to be a hallmark of PD and is closely related to mitochondrial function [30]. Mitochondrial function can be reflected by the changes in mitochondrial transmembrane potential, the collapse of which further induces ROS overproduction [31]. The disruption of complex I activity and mitochondrial transmembrane potential lead to the reduction of ATP production. In the current study, we demonstrated that rotenone induced the overproduction of ROS, which is effectively attenuated by RA. Rotenone downregulated mitochondrial membrane potential and ATP level, while RA treatment leads to the partial restoration of them. These findings suggested that RA reversed the mitochondrial function reduction induced by rotenone in SH-SY5Y cells.

One of the characteristics of PD is the abnormal accumulation and aggregation of α-synuclein in the form of Lewy bodies [32]. α-synuclein induces neuronal toxicity and death through causing mitochondrial dysfunction, lysosomal impairment, and membrane disturbance [33]. Pathological aggregation and phosphorylation of Tau protein at Ser404 are also involved in many neurodegenerative diseases including PD [34]. Studies have shown that PD patients with cognitive impairment have elevated level of phosphorylated Tau in Lewy bodies along with α-synuclein [35]. In the current study, rotenone caused overexpression of α-synuclein and hyperphosphorylation of Tau, and RA markedly reduced α-synuclein expression and Tau phosphorylation. These results implied that RA protected neuronal cells potentially through suppression α-synuclein accumulation, aggregation, and reduction of Tau protein phosphorylation.

Disruption of mitochondrial biogenesis has been shown to be associated with PD development. The peroxisome proliferator-activated receptor gamma coactivator 1 (PGC-1α) is considered as a master regulator of mitochondrial biogenesis and antioxidant response [36]. PGC-1α function is down-regulated in PD [37]. PGC-1α knock out mice developed higher vulnerability to the neurodegenerative effects of MPTP [38]. Serine/threonine kinase AMPK is activated by falling energy levels and plays an important role in restoring cellular energy balance [25]. Activating AMPK has multiple effects in PD model including changing cellular metabolism, promoting autophagy, enhancing mitochondrial quality control, and increasing antioxidant capacity [25]. AMPK also promotes mitochondrial biogenesis and reduces ROS through activation of PGC-1α [39]. Several studies showed that AMPK activation facilitated the clearance of α-synuclein and promoted neuronal survival [40]. Akt signaling is central to cell survival and is impaired in PD [26]. Selective loss of dopaminergic neurons was accompanied by a decrease of Akt phosphorylation at Ser473 in the PD brain [41]. In the current study, PGC-1α protein and Akt phosphorylation levels were evidently reduced in the presence of rotenone, RA led to restorations of PGC-1α expression and Akt phosphorylation. Rotenone induced an elevation of AMPK Thr172 phosphorylation, while RA treatment further promoted AMPK Thr172 phosphorylation. The initial upregulation of AMPK phosphorylation induced by rotenone could be due to the cellular self-adapting mechanism. Under stress conditions, cells would initially activate certain pro-survival signaling pathways including AMPK to attempt to overcome the encountered stress condition [42]. These observations highlighted that RA restored the protein expression and phosphorylation of the prosurvival, neuroprotective, and mitochondrial function-promoting proteins in rotenone induced PD cell model.

Multiple animal models of PD suggested that activation of Abl played an important role in the initiation and progression of neurodegeneration [1]. To investigate if the suppression of Abl is involved in the neuroprotective effect of RA against rotenone, we analyzed the tyrosine phosphorylation level of Abl Tyr412, which is located at the kinase activation loop of Abl. The phosphorylation of this site is required for Abl kinase activation [43]. In the present study, rotenone markedly induced Abl Tyr412 phosphorylation. RA treatment suppressed this hyperphosphorylation induced by rotenone, which mimicked the effect of Abl specific inhibitor, nilotinib. Nilotinib treatment also partially restored SH-SY5Y cell viability reduced by rotenone. To further assess the activity of Abl, the phosphorylation state of an Abl substrate, CrkII, was analyzed at its Abl phosphorylation site Tyr221 [44]. Rotenone exposure increased CrkII Tyr221 phosphorylation, RA treatment suppressed this hyperphosphorylation induced by rotenone. Like RA, nilotinib also significantly restored cell viability in rotenone exposed SH-SY5Y cells. These results suggested that RA exerted the protective effect on SH-SY5Y cells partially through the suppression of Abl activation. Moreover, Abl inhibition with nilotinib also had similar effects to that of RA on the regulation of the phosphorylation state and protein expression level of several key signaling proteins involved in PD and mitochondrial function regulation. Nilotinib reduced the protein level of α-synuclein, phosphorylation level of Tau Ser404, and promoted phosphorylation of Akt Ser473, AMPK Thr172, and expression of PGC-1α. Both nilotinib and RA treatment effectively suppressed rotenone induced ROS overproduction and increased mitochondrial membrane potential and cellular ATP level reduced by rotenone. These observations indicated that at least part of the neuroprotective function of RA is due to its inhibitory effect on Abl. Nilotinib alone reduced CrkII Tyr221 phosphorylation and did not lead to any significant changes in Abl Tyr412 phosphorylation. This suggests that the basal activity of Abl is low, and it is slightly further inhibited by nilotinib. However, since this activity reduction is small, it did not further modify the ROS, mitochondrial membrane potential, ATP level and the phosphorylation of other PD-related signaling proteins.

As a mitochondrial complex I inhibitor, rotenone causes mitochondrial dysfunction and overproduction of ROS [18]. ROS activates Abl protein, which causes accumulation of α-synuclein, further disrupts mitochondrial function and eventually leads to neuronal cell death [4]. In addition to α-synuclein, studies have shown that Abl also phosphorylates and inactivates an E3 ubiquitin ligase, Parkin [45]. Since Parkin interacts with Pink1 and plays an important role in the clearance of dysfunctional mitochondria, cell death mediating protein PARP-1 and other unwanted intracellular proteins, the inactivation of Parkin leads to the loss of neuronal cells [16]. Abl can phosphorylate PKC [46]. PKC activation leads to the phosphorylation of AMPK Ser487, which results in the reduction of AMPK activity [47]. Thus, inhibition of Abl could possibly increase AMPK activity through this pathway and promote cell survival. This explains the reason for which nilotinib treatment alone induced AMPK Thr172 phosphorylation in our experiment. Moreover, Abl activation also leads to dopaminergic neuron loss by phosphorylating and activating p38α MAPK [48]. In the current study, we speculate that RA inhibits Abl protein signaling by reducing the intracellular ROS level. However, further study is needed to investigate other potential pathways through which RA leads to the inhibition of Abl.

## 5. Conclusions

In conclusion, this is the first study indicating that natural compound RA attenuated the neurotoxicity induced by rotenone in SH-SY5Y Parkinson’s disease cell model through inhibiting Abl and ameliorating the mitochondrial dysfunction. This study also demonstrated that downregulation of α-synuclein protein, Tau phosphorylation, and increases of signaling proteins Akt Ser473, AMPK Thr172 phosphorylation and PGC-1α level were involved in the neuroprotective effect of RA. This finding contributes to a new understanding with regard to the molecular mechanisms underlying the neuroprotective effect of RA in PD and will promote the further use of RA as a natural neuroprotective reagent.

## Data Availability

Not applicable.
